# Empathy and attitude toward communication skill learning as a predictor of patient-centered attitude: a cross-sectional study of dental students in Korea

**DOI:** 10.1186/s12909-021-02674-z

**Published:** 2021-04-21

**Authors:** Minjung Lee, Jungjoon Ihm

**Affiliations:** 1 Department of Public Health Sciences, Graduate School of Public Health, Seoul National University, Seoul, South Korea; 2 Office of Dental Education, School of Dentistry, Seoul National University, Seoul, South Korea; 3 Dental Research Institute, School of Dentistry, Seoul National University, Seoul, South Korea; 4 Interdisciplinary Program in Cognitive Science, Seoul National University, Seoul, South Korea

**Keywords:** Patient-centered care, Patient-centered attitudes, Empathy, Communication skills attitudes, Dental students

## Abstract

**Background:**

Enhancing medical students’ practice of patient-centered care is a goal of medical schools. In addition to exploring the demographic and academic factors of the students, it is necessary to identify other attitudes and perceptions that may influence the student’s patient-centered attitude and inclination toward communication skill learning. This study aimed to assess patient-centered attitudes among dental students in Korea and identify the association between the students’ characteristics and empathy, communication skill learning attitude, and patient-centered attitude.

**Methods:**

Data were collected via a cross-sectional online survey, and 312 dental students were included in the analyses. The study participants completed the Patient–Practitioner Orientation Scale (PPOS), the Interpersonal Reactivity Index (IRI), and the Communication Skills Attitude Scale (CSAS). Analyses were performed using independent samples’ t-tests, hierarchical multi-variable regression, and ANOVA with a post-hoc Tukey test.

**Results:**

The students tend to be moderately patient-oriented toward the sharing subscale of PPOS score (M = 3.78, standard deviation [SD] = 0.54) and slightly more patient-centered toward the caring subscale of PPOS score (M = 4.41, SD = 0.52) of patient-centered attitudes. Being a female and a shorter academic period in dentistry were associated with attitudes toward patient-centered care. Empathy and positive attitude toward learning communication skills were also related to a patient-centered attitude, and among aspects of empathy, “empathic concern” had the greatest significant impact on patient-centered attitude.

**Conclusions:**

Gender, academic period, empathy, and attitudes on learning communication skills were important influencing factors of patient-centered attitudes. Patient-centered attitude can and must be taught. Education programs should focus on enhancing empathy, emphasizing positive attitudes on learning communication skills, and conducting follow-up educational sessions to prevent students from becoming less patient-centered with an increase in duration of their academic period.

**Supplementary Information:**

The online version contains supplementary material available at 10.1186/s12909-021-02674-z.

## Background

Patient-centered care that aims to identify and respond to thoughts and emotions related to the patient’s health problems and to establish a common ground for the patient’s health problems, treatment, and the respective roles are recognized by both the patients and doctors [1]. In recent decades, increasing evidence has suggested that patient-centered care engenders positive outcomes, including higher patient satisfaction, increased adherence to medication and behavioral regimens, higher healthcare provider satisfaction, and superior clinical outcomes [2–5]. Moreover, patient-centered care is a medical principle that has been discussed most frequently in the previous few decades [6]. Currently, in Korea, there is an active movement to create a patient-centered healthcare system and provide patient-centered care. The Ministry of Health and Welfare in Korea is proposing a policy goal to provide patient-centered medical services, and it is rapidly spreading via the performance of Patient Experience evaluation since 2017 [7] and public health policy initiatives such as “in-depth consultations of over 15 minutes” health services for patients with serious and non-curable diseases since 2018 [8]. However, as per a recent Organisation for Economic Co-operation and Development (OECD) Health Statistics report, the number of outpatient visits in Korea is among the highest among OECD countries (16 times a year), 87.1% of the patients easily understood the doctor’s explanation (13th among 19 countries), and 81.8% had the opportunity to speak up with questions or concerns to the healthcare provider (13th among 18 countries), the lowest among OECD countries [9]. Over 80% of patients were satisfied, but there is still room for improvement. It is vital to educate and train medical and dental students to provide patient-centered care.

Enhancing medical students’ practice of patient-centered care is a goal of medical schools [10, 11]. To promote the benefits of both patients and healthcare providers, educational programs for healthcare professionals in many countries are increasingly being adopted in the curriculum that advocates patient-centered approach. The curriculum also aims to respond to a decline in the patient-centered attitudes in the medical curriculum. Although evidence on patient-centered attitudinal change during the course of medical education remains contradictory, some studies have reported either an increase or no significant decline [12, 13]; however, a decline in patient-centered attitudes has been observed in longitudinal studies in the USA, Japan, and Greece and has been inferred from several cross-sectional studies internationally [14–16]. The patient-centered attitude of medical students remained at a certain level during the preclinical stage [17, 18]; however, this was evidenced to witness a decline during the clinical years [19, 20]. Moreover, gender-based differences have been consistently reported in previous studies, with female students generally exhibiting a more patient-centered attitude than male students [12, 14, 21, 22]. A previous meta-analysis of observational studies of physician communication indicated that female physicians engaged in more communication that could be considered patient-centered than their male counterparts [23]; therefore, gender-based differences in attitudes toward physician–patient relationships might have started at the stage of medical education. In this regard, infusing patient-centeredness into medical school curricula could help future healthcare providers deliver quality care and build effective health systems; however, it requires an understanding of the existing levels and patterns of attitudes toward patient-centeredness [24].

In addition to exploring the demographic and academic factors relating to the students, it is necessary to identify other attitudes and perceptions that may affect the student’s patient-centered attitude. Empathy, an essential characteristic that is commonly included in many different definitions of the concept of patient-centered care [25, 26], can be a potential factor that affects patient-centered attitude. Empathy can be defined as “the ability to understand the patients’ situation, perspective, and feelings, and to communicate that understanding to the patient” [27]; it is an essential component of both, competence as a doctor and the physician–patient relationship [28, 29]. In particular, empathy has been suggested to strengthen the therapeutic alliance between the healthcare provider and patient and could be a pathway through which patient-centeredness impacts patients and, ultimately, patient outcomes [5]. Few studies have found a positive association between empathy and patient-centered attitude among medical students [30] and among dental medicine students [31]. More importantly, it is found that experiences can modify empathy and can be learned through a proper educational program during medical school. Increasing evidence has demonstrated positive results for several educational programs designed to improve empathy among medical students, residents, and doctors [32, 33]. According to a study investigating longitudinal change of empathy among Polish dental students using Jefferson Scale of Empathy – Health Profession Students Version (JSE-HPS), gender related changes of empathy level over the different stages in their training course was observed [34]. Therefore, empathy might be a potential element in fostering patient-centered attitude through education.

Another factor expected to contribute to patient-centered attitude is attitude toward communication skill learning. Communication skills with patients and other healthcare practitioners are essential for patient-centered clinical practice and represent an important medical competence of doctors. Rees, Sheard, and Davies published the Communication Skills Attitude Scale (CSAS) that measures students’ attitudes toward learning communication skills during medical school [35]. Previous studies have reported that female students have more positive attitude than their male counterparts, and students in their initial years have a more positive attitude than those who are in their later years of education in medical school [36, 37]. Student’s positive or negative evaluations of learning communication skills may influence patient-centered attitudes. To our knowledge, no study has examined the effect of communication skills learning on patient-centered attitudes.

The concept of patient-centered care is becoming increasingly prominent in the field of dentistry. However, despite the growing importance of this concept, there is lack of research on the attitude toward patient-centeredness among dental students and on how it can be inculcated and practiced in clinical settings [38]. Specifically, this study aims to (1) investigate attitudes toward patient-centered care of dental students in Korea in terms of patient vs. doctor-centered attitudes, (2) identify student’s characteristics that are associated with patient-centered attitudes, and (3) examine the influence of empathy and attitudes toward learning communication skills on patient-centered attitudes. Moreover, this study also discusses complications in the development and implementation of a patient-centered curriculum in dental education.

## Methods

### Participants

This study was approved by the Institutional Review Board of the School of Dentistry, the Seoul National University, Seoul, Republic of Korea, as per the policy on research with human participants (Institutional Review Board No. S-D20200028). The participants of this cross-sectional study were 312 dental students enrolled at the Seoul National University School of Dentistry, Seoul, Republic of Korea for the spring 2020 semester. These participants completed the Patient–Practitioner Orientation Scale (PPOS), the Interpersonal Reactivity Index (IRI), and the CSAS. All the respondents had provided informed consent for their data to be included in this study.

### Data collection

A cross-sectional survey was designed to evaluate the dental students’ patient-centered orientation, empathy, and communication skills learning attitude using an anonymous online questionnaire. The survey was conducted via an online platform with Google survey. The Korean Government has asked the general public to minimize face-to-face interaction and isolate themselves at home; therefore, potential respondents were electronically invited. They completed the questionnaires in Korean via an online survey platform. All the respondents provided informed consent. The process of data collection occurred over a period of 6 weeks (June 2–June 15, 2020).

### Measurements

#### Patient–practitioner orientation scale (PPOS)

The study participants completed the PPOS, an 18-item self-report measure that uses two subscales, “caring” (i.e., understanding patients’ perspectives) and “sharing” (i.e., sharing responsibilities and power in decision-making with patients) to quantify the roles that healthcare practitioners and patients believe that each should play in the process of their interaction along a doctor-centered/patient-centered continuum [15, 39]. The PPOS questionnaire was translated into the Korean language, was modified as per the Korean healthcare context, and was validated [40, 41]. Some samples include the following: “Patients should be treated as if they are partners of the doctor, equal in power, and status” (Sharing subscale), “When doctors ask a lot of questions about a patient’s background, they are prying too much into personal matters” (Caring subscale). The responses were rated on six-point Likert scale, with “1=strongly agree to 6= strongly disagree.”

#### Interpersonal reactivity index (IRI)

In this study, the Korean version of the IRI (K-IRI) that was translated and validated by Kang et al. [42] was used. This K-IRI has been used to assess empathy among Korean medical students and is found to have adequate psychometric properties [43]. The IRI was designed to measure individual differences in empathy among the general population. It includes 28 items with response options on a five-point Likert-type scale (0 = does not describe me very well, 4 = describes me very well). The IRI has a multidimensional approach of empathy, which can be measured by two cognitive subscales: 1) Perspective taking (IRI-PT) that measures the tendency to spontaneously adopt the psychological viewpoint of others and 2) Fantasy (IRI-FS) that measures the ability to transform oneself into characters of movies, books, plays, etc., and affective aspects, which can be measured using two affective subscales: 3) Empathic concern (IRI-EC) that measures the ability to assess “other” oriented feelings of sympathy and concern for unfortunate others and 4) Personal distress (IRI-PD) that measures “self” oriented feelings of distress and unease in interpersonal settings, using a 28-item Likert scale questionnaire.

#### Communication skills attitude scale (CSAS)

The CSAS was designed to measure the attitude toward communication skill learning [35], and it is most specific to teaching and learning of communication skills and is the most widely used and validated scale [44]. The CSAS comprises of a scale that is divided into two aspects, which includes positive and negative attitudes toward learning communication skills and comprises 26 items—each with 13 items. The contents of the subscale include the Positive Attitudes Scale (CSAS-PAS) that are positively worded (e.g., “In order to be a good doctor I must have good communication skills”) and the Negative Attitudes Scale (CSAS-NAS) that are negatively worded (e.g., I do not need good communication skills to be a doctor). The measurement uses a five-point Likert scale (1 = strongly disagree, 5 = strongly agree). The scores for every scale ranged from 13 to 65, with higher scores revealing stronger positive or negative attitudes toward learning communication scale.

### Data analyses

All statistical analyses were performed using R version 3.5.1 (R Foundation for Statistical Computing, Vienna, Austria). All the results of quantitative variables were reported either as mean (M), standard deviation (SD), or frequency (percentage %). Independent t-test was used to compare the differences in the PPOS scores, IRI scores, and CSAS scores of male and female dental students. Multivariate hierarchical linear regression analysis was used to examine the effect of sociodemographic aspects, academic factors, and empathy, and attitude related to communication skills on patient-centered orientation. ANOVA with a post-hoc Tukey test was employed to examine the average differences among the student’s future career plans with regard to patient-centered attitude.

## Results

### Participants’ demographic and education-related characteristics

We collected surveys from 312 students that included 177 males (56.7%) and 135 females (43.3%), with a mean age of 22 years (M = 22.39, SD = 3.02) (Table 1). The study school has been operating as a dental college (3 years pre-dental course and 4 years Doctor of Dental Surgery [DDS] degree program) and a professional graduate-entry school (4 years DDS degree program) system simultaneously. Students who enter the professional graduate-entry school are required to have a bachelor’s degree in advance, while students who enter the dental college are mostly high school graduates. Students from both tracks merge and work together to attain the level of a DDS degree. Among the study participants, 61.9% were from the *dental college*, and 38.1% belonged to *professional graduate-entry school*. The average academic duration was 2.92 y (SD = 1.77). The students’ demographic and academic characteristics are presented in Table 1.

**Table 1:** Descriptive statistics of survey respondents (*n* = 312)

Characteristics	No.	%
**Gender**		
Male	177	56.7
Female	135	43.3
**Age groups**	M^a^ = 22.39	SD^b^ = 3.02
≤ 25 y	261	83.7
> 25 y	51	16.3
**Academic track**		
Dental college	193	61.9
Professional graduate-entry school	119	38.1
**Academic period**	M = 2.92	SD = 1.77
≤ 3 years	221	70.8
> 3 years	91	29.2
**Future career plans**		
Private dental practices (self-employed)	158	50.60%
Private dental practices (employed)	39	12.50%
University hospital	87	27.90%
Other/Don’t know	28	9.00%

^a^*M* Mean

^b^*SD* Standard Deviation

### Patient-centered attitude, empathy, and communication skills learning attitudes

The mean PPOS score for the entire sample was 4.09 (SD = 0.45), near the midpoint of the possible range (1–6). The mean score was slightly lower for the sharing subscale 3.78 (SD = 0.54) and slightly higher for the caring subscale 4.41 (SD = 0.52) (Table 2). Cronbach’s alpha was high for the full scale (α = 0.72) and had an acceptable level for the caring subscale (α = 0.59) and the sharing subscale (α = 0.61). Higher PPOS values indicate more patient-centered attitude in terms of sharing power with patients and holistic patient care.

**Table 2:** Descriptive statistics for Patient–Practitioner Orientation Scale, empathy, and communication skills learning attitude

Variable	M	SD
Patient–Practitioner Orientation Scale (PPOS^a^)		
PPOS (Sharing)	3.78	0.54
PPOS (Caring)	4.41	0.52
PPOS (Total)	4.09	0.45
Empathy (IRI^b^)		
IRI (Fantasy)	3.52	0.62
IRI (Empathic Concern)	3.66	0.52
IRI (Perspective Taking)	3.69	0.5
IRI (Personal Distress)	2.95	0.51
Communication Skills Attitude Scale (CSAS^c^)		
Positive Attitudes Scale (CSAS-PAS^d^)	4.09	0.61
Negative Attitudes Scale (CSAS-NAS^e^)	2.59	0.93

^a^*PPOS* Patient–Practitioner Orientation Scale

^b^*IRI* Interpersonal Reactivity Index

^c^*CSAS* Communication Skills Attitude Scale

^d^*CSAS-PAS* Communication Skills Attitude Scale – Positive Attitude Scale

^e^*CSAS-NAS* Communication Skills Attitude Scale – Negative Attitude Scale

Descriptive analysis was performed on the four subscales of empathy, and the results are presented in Table 2. Among the four subscales, perspective taking score was the highest (M = 3.69, SD = 0.50), followed by empathic concern (M = 3.66, SD = 0.52), fantasy (M = 3.52, SD = 0.62), and personal distress (M = 2.95, SD = 0.51). Cronbach’s alpha was high for the full scale (α = 0.77), the perspective taking subscale (α = 0.64), empathic concern (α = 0.68), fantasy (α = 0.75), and the personal distress (α = 0.66). The mean value of the CSAS showed that the students have positive attitudes toward learning communication skills (Table 2).

The CSAS-PAS score (M = 54.15, SD = 6.36) was higher than the CSAS-NAS score (M = 32.65, SD = 5.96) (Table 2). Among the CSAS-PAS items, “Learning communication skills has helped or will help me respect patients.” (M = 4.51, SD = 0.61) had the highest score, and among the CSAS-NAS items, “I haven’t got time to learn communication skills” (M = 3.29, 1.02) had the highest score. Cronbach’s alpha was high for CSAS-PAS scale (α = 0.88) and CSAS-NAS scale (α = 0.71).

### Gender-based differences in patient-centered attitudes, empathy, and communication skills learning attitudes

By examining the patterns of PPOS score, IRI, and CSAS, we assessed whether gender-based differences exist. We found significant differences between female and male students. Female students had a significantly higher overall PPOS score and sharing subscale than male students. The score of the female students’ PPOS caring subscale was higher than that of the male students; however, the difference was not statistically significant. With respect to IRI, fantasy, empathic concern, and personal distress had higher scores among female students and perspective taking was higher in males. However, only the differences for personal distress were significant between males and females (*p* < 0.001). Moreover, there was no significant gender-based difference in the CSAS-PAS and CSAS-NAS (Table 3).

**Table 3:** Comparison of male and female dental student scores on the Patient–Practitioner Orientation Scale (PPOS), Interpersonal Reactivity Index (IRI), and Communication Skills Attitude Scale (CSAS)

Instrument	Item group	n	M^d^	SD^e^	t-value	Degree of freedom	***p***-value	Mean difference	95% confidence interval	
									Lower	Upper
PPOS^a^	Sharing	Male = 177	3.72	0.54	−2.38	310	0.02	−0.15	−0.26	− 0.03
		Female = 135	3.86	0.52						
	Caring	Male = 177	4.38	0.56	−0.99	310	0.32	− 0.06	− 0.18	0.06
		Female = 135	4.44	0.46						
	Total	Male = 177	4.05	0.48	−1.99	310	0.04	−0.1	−0.2	0
		Female = 135	4.15	0.4						
IRI^b^	Fantasy	Male = 177	3.47	0.65	−1.65	310	0.1	−0.12	− 0.26	0.02
		Female = 135	3.59	0.59						
	Empathic concern	Male = 177	3.63	0.56	−1.19	310	0.24	−0.07	− 0.19	0.05
		Female = 135	3.7	0.46						
	Perspective taking	Male = 177	3.72	0.53	1.13	310	0.26	0.06	−0.05	0.18
		Female = 135	3.65	0.45						
	Personal distress	Male = 177	2.88	0.52	−2.85	310	0	−0.17	−0.28	−0.05
		Female = 135	3.05	0.49						
CSAS^c^	Positive	Male = 177	53.86	6.38	−0.92	289.63	0.36	−0.67	−2.1	0.76
		Female = 135	54.53	6.33						
	Negative	Male = 177	32.94	6.12	1	296.61	0.31	0.68	−0.65	2.01
		Female = 135	32.26	5.76						

^a^*PPOS* Patient–Practitioner Orientation Scale

^b^*IRI* Interpersonal Reactivity Index

^c^*CSAS* Communication Skills Attitude Scale

^d^*M* Mean

^e^*SD* Standard Deviation

### The effect of empathy and communication skills learning attitude on patient-centered attitudes

Influencing factors on patient-centered attitudes from among demographic factors, academic factors, empathy, and communication skills learning attitude were assessed (Table 4). The investigated factors accounted for about 31.3% of the variance in the total PPOS score, F (9, 302) = 16.72, adjusted R^2^ = 0.31, *p* < 0.001. Gender (female) (β = 0.10, *p* = 0.04), academic period (β = − 0.03, *p* = 0.03), and empathic concern (β = 0.19, *p* < 0.001) were significant individual predictors of the overall PPOS score. Both, positive and negative attitudes about learning communication skills were related to the overall PPOS score. Admission type and other subscales of empathy except empathic concern did not influence the total PPOS score. Similar to the overall PPOS score, gender (female) (β = 0.15, *p* = 0.01), academic period (β = − 0.06, *p* < 0.001), and empathic concern (β = 0.16, *p* = 0.02) were significant variables that influenced the PPOS sharing subscale. However, unlike the overall PPOS score, only negative attitude of learning communication skills (β = − 0.31, *p* < 0.001) was associated with the sharing subscale of the PPOS score. Among the influencing factors, the effect of empathic concern was the strongest. The investigated factors accounted for approximately 17.9% of the variance in the sharing subscale of the PPOS score. Finally, for the PPOS caring subscale, none of the demographic factors were significant. Empathy (fantasy) (β = 0.13, *p* = 0.01), empathic concern (β = 0.22, *p* < 0.001), and personal distress (β = − 0.12, *p* = 0.04) were significant individual predictors of the PPOS caring subscale. Moreover, both, positive and negative attitudes on the communication scale were related to the PPOS caring subscale. The investigated factors accounted for about 31.0% of the variance in the caring subscale of the PPOS score.

**Table 4:** Multivariate hierarchical linear regression analysis of factors on patient-centered attitudes from among demographic and academic factors, empathy, and communication skills learning attitude

Variable		PPOS^**a**^ (Total)			PPOS (Sharing)			PPOS (Caring)		
		B	Beta	p	B	Beta	p	B	Beta	p
Step1	Gender (Male:1, Female:2)	0.1	0.11	0.04	0.15	0.14	0.01	0.05	0.05	0.34
	Academic track (DC^b^:1, Professional:2)	−0.02	− 0.02	0.73	−0.06	−0.06	0.35	0.03	0.02	0.68
	Academic Period	−0.03	−0.13	0.03	−0.06	−0.21	< 0.001	0	−0.01	0.91
	Adjusted R-squared	0.05			0.06			0.03		
Step2	IRI^c^ (Fantasy)	0.08	0.11	0.08	0.03	0.03	0.61	0.13	0.16	0.01
	IRI (Empathic Concern)	0.19	0.22	< 0.001	0.16	0.15	0.02	0.22	0.22	< 0.001
	IRI (Perspective Taking)	0.08	0.09	0.14	0.09	0.09	0.19	0.08	0.07	0.23
	IRI (Personal Distress)	−0.07	−0.09	0.14	− 0.03	− 0.03	0.6	−0.12	− 0.12	0.04
	Adjusted R-squared	0.18			0.1			0.19		
Step3	^d^CSAS_PAS	0.01	0.13	0.04	0	0.03	0.69	0.02	0.19	< 0.001
	^e^CSAS_NAS	−0.03	−0.34	< 0.001	−0.03	−0.31	< 0.001	−0.03	−0.28	< 0.001
	Adjusted R-squared	0.31			0.18			0.31		

^a^*PPOS* Patient–Practitioner Orientation Scale

^b^*DC* Dentistry College

^c^*IRI* Interpersonal Reactivity Index

^d^*CSAS_PAS* Communication Skills Attitude Scale (Positive)

^e^*CSAS_NAS* Communication Skills Attitude Scale (Negative)

### Differences in patient-centered attitudes according to students’ future career plans

We also investigated the patterns of patient-centered attitudes as per the future career plans of the students. About 50% of the students wanted to be self-employed in private dental practices (50.6%), followed by those who wanted to work in university hospitals (27.9%), those who wished to get employed at private dental practices (12.5%), and those who were unsure of their future (9.0%). When we compared the PPOS score among those with different future career plans, students who planned to be employed in private dental practices or university hospitals showed significantly higher scores overall and in sharing subscale of PPOS than students who planned to be self-employed (Table 5).

**Table 5:** Patient-centered attitude among medical practitioners with different future plans

	n (%)	PPOS^a^ (total)		PPOS (sharing)		PPOS (caring)	
		Mean	SD^b^	Mean	SD	Mean	SD
Private dental practices (self-employed)	158 (50.6%)	3.98_a_	0.41	3.66_a_	0.48	4.31	0.5
Private dental practices (employed)	87 (27.9%)	4.20_b_	0.48	3.90_b_	0.57	4.5	0.52
University hospital	39 (12.5%)	4.23_b_	0.4	3.93_b_	0.55	4.53	0.49
Other/Don’t know	28 (9.0%)	4.19	0.52	3.9	0.6	4.49	0.57
F	312	6.68***		5.94***		3.77**	

^a^*PPOS* Patient–Practitioner Orientation Scale

^b^*SD* Standard Deviation

Notes: Significant difference in post-hoc Tukey test at alpha = 0.05: a < b, ***p < 0.001, **p < 0.01

## Discussion

Our study results provide valuable insights into dental students’ attitudes toward patient-centered care and identify its association with students’ characteristics, empathy, and attitudes on learning communication skills. Students tend to be more patient-centered in the caring subscale than in the sharing subscale. Being a female and having a shorter academic period in the field of dentistry, higher level of empathy, and positive attitudes toward learning communication skills were associated with more patient-centered attitudes. “Empathic concern” aspect of empathy had the highest and a significant impact on the patient-centered attitude.

Several interesting findings worth noting exist. First, students preferred a slightly more patient-centered role in the caring aspect (4.41 ± 0.52) than in the sharing aspect (3.78 ± 0.54). For example, students were most patient-centered (4.97 ± 1.01) for the statement “If doctors are truly good at diagnosis and treatment, the way they relate to patients is not that important (caring).” The lowest mean score (2.54 ± 0.87) signifying most doctor-centered attitude was received for “The patient must always be aware that the doctor is in charge (sharing).” However, the inferences from the subscale and overall PPOS scores rather than those of individual items are more appropriate in the current study. Compared with that in previous studies, the overall PPOS score (4.09 ± 0.45) was higher than the mean score reported among Indian dental students (3.38) [22] and Korean (3.90) [45] medical students; however, it was lower than that reported among dental students (4.37) in the USA [46] and medical students in the USA (4.57), Japan (4.56) [47], and Brazil (4.66) [48]. Scores from our study and those from previous studies should be interpreted with caution because the internal consistency was either inadequate or not reported in the publications. Nevertheless, these results indicate that efforts to improve patient-centered attitudes among dental students are needed.

Second, the current study demonstrated that students’ background characteristics (i.e., gender and academic period in the field of dentistry) were associated with patient-centered attitudes. Consistent with previous studies [12, 14, 21, 22], this study also displayed that female students had a more patient-centered attitude than their male counterparts. The influence of gender was more evident for the sharing aspect than the caring aspect of patient-centered care. Considering evidences of female physicians practicing more patient-centered care than their male counterparts, [23] this result suggests that gender-based differences in attitudes toward physician–patient relationships might have started at the stage of medical education. Patient-centered attitudes of students were maintained after graduation and influence the patient-centeredness of the practicing style in clinical settings. When it comes to students’ academic background, a longer academic period has been identified as a significant factor that lowers the patient-centered attitudes. Although evidence on patient-centered attitudinal change during the course of medical education remains contradictory, various studies have shown that the attitudes of students in the later years of medical school are more doctor-centered or paternalistic than those among students during the initial years [14, 15, 47]. Although the design of this study is cross-sectional, these results provide evidence that patient-centered erosion might occur among Korean dental students. Reductions in patient-centered attitudes may result from the considerable emphasis that medical programs place on the biological aspects of the disease and the emotional burn-out medical students may develop as their responsibilities and workload intensify [24]. These results also suggest that it will be very challenging to develop an effective curriculum that improves the patient-centered attitude of Korean dental students and maintains it after graduation. Medical curricula should not be “one-size-fits-all.” Targeting students’ gender and academic period differences with specific interventions is needed in curriculum, professional development programs, and patient simulations.

Third, empathy was a key contributor of the patient-centered attitude of dentistry students, which was in line with earlier studies [46, 49]. This study measured students’ empathy using the IRI, which is comprehensively determined in that it examines four individual aspects of empathy comprising both cognitive and affective aspects [50–52]. Therefore, the effect of each of the four individual aspects of empathy on patient-centered attitude was examined.

Empathy was the most important predictor of patient-centered attitudes in terms of both sharing and caring because it explained 4.8% and the 18.1% of the sharing and caring variance, respectively. For the “sharing” aspect of patient-centered attitudes, an affective aspect of empathy, “empathic concern” was the only significant predictor among the aspects of empathy. According to Hojat and his colleagues, empathic concern is an affective component of empathy that assesses “other-oriented” feeling of sympathy and concern for unfortunate others and is more relevant to patient care situations than other aspects [52, 53]. The ability to share the patients’ feelings and concerns can help students agree with the idea of sharing aspects of patient-centered care—that patients should be treated as partners with the doctor with equal power and status and that doctors should share information and try to share responsibility in decision-making.

When it comes to the caring aspect of patient-centered attitude, it was related to not only affective but also cognitive aspect of empathy. Not only “empathic concern” but also “fantasy” and “personal distress” aspects of empathy were positively associated with it significantly. “Fantasy” refers to the extent to which the respondents transpose themselves imaginatively into the feelings and actions of others [54], which are likely to appraise the cognitive component of empathy. This result suggests that the cognitive ability to imagine and engage a patient’s feelings is related to being open and warm and fostering therapeutic relationships with the patients and caring about the patient’s expectations, preferences, and emotions. Interestingly, higher “personal distress” has demonstrated a negative impact on patient-centered attitudes. The personal distress aspect of empathy refers to the extent of an individual’s feelings of anxiety and discomfort owing to a negative experience [50]. The affective aspect of this empathy may relate to the student’s preference for more instrumental interactions rather than psychosocial interactions with patients as well as doctor-centered roles in relationships with patients. This is consistent with previous studies showing that medical students with high scores in personal distress prefer limited exposure to affectively charged situations and less contact or interaction with patients [50, 55, 56]. Moreover, too much personal distress of empathy can sometimes produce higher levels of burn-out, termed as “compassion fatigue” [57, 58].

Finally, attitudes regarding learning communication skills were a significant factor that affected the students’ patient-centered attitude; negative attitudes were related to doctor-centered attitude, and positive attitudes were related to patient-centered attitude. Therefore, teaching and emphasizing the importance of communicating with patients can help students develop a more patient-centered attitude toward caring and sharing. For example, efforts to make students believe that learning communication skills can help enhance their practice and facilitate them to provide enough time to learn communication skills can be a practical and efficient way to enhance the patient-centeredness of the student’s attitude on physician–patient relationships.

In addition, a significant difference exists in patient-centered attitude between students who planned to be self-employed in private practices and employed in private practices or university hospitals. Students who planned to be self-employed have shown relatively doctor-centered attitude than students who planned to be employed in private or university hospitals. Although they have a commonality that they provide dental services to patients, self-employed dentists reported more autonomy and control than organizationally employed dentists [59, 60]. Students’ attitudes toward and reasons for choosing careers are of great interest for educational systems and this study suggests that patient-centered attitude should be investigated as one of the potential determinants of student’s future career choice.

This study has certain limitations. First, the data of this study was obtained via an online survey tool. Despite the benefits of online surveys (e.g., higher response rates, lower costs, and anonymity) a risk for falsification exists due to less responsibility and the absence of interviewers. The second limitation is that the participants of our study were from only one Korean dental school; therefore, generalization of the present results is limited. A possible suggestion for future studies is the use of a multicentric research design. Third, this was a cross-sectional and not a longitudinal study, making it necessary to treat comparisons between academic periods with caution, which applies for most of the earlier studies as well.

### Implication of this study

A set of implications for education and future research can be drawn from the findings of this study. First, various educational interventions targeted toward increasing dental students’ empathy can be effective in enhancing the patient-centered attitude of medical and dental students. Previous studies suggested students’ reflection on how effective their interviews with simulated patients were in terms of empathic communication, and project-based learning approach helps students in developing their empathy [43, 49]. Second, this study suggests that students with low empathic concern and high personal distress may need intensive support. This is because this type of student might tend to have doctor-centered attitude, and higher risk for burnout [58]. The current study also supports that education programs should focus on emphasizing positive attitudes regarding learning communication skills and facilitate them to provide enough time for learning communication skills. Third, longitudinal research on the dynamics of patient-centered attitude during academic periods of dental students should be investigated. Longitudinal monitoring of empathy is investigated in future studies because students’ level of empathy has been found to be an important determinant of patient-centered attitude and has been found to change across the academic years [34, 61, 62].

## Conclusions

In conclusion, female students’ attitudes toward patient-centered care were more patient-centered than their male counterparts; the attitudes of students in the later years of medical school were more doctor-centered than those of students during the initial years. Our study suggests that the type of empathy that students manifest contributes to the PPOS score in terms of the role they prefer in patient-physician relationships in both sharing and caring. In this study, empathic concern and fantasy type of empathy tended to be associated with more patient-centered attitudes, while personal distress type of empathy was associated with more doctor-centered attitude. Attitudes on learning communication skills were also an important influencing factor of patient-centered attitudes. Importantly, attitudes toward patient-centered care must be taught, and can be learned. The findings of this study provide useful insights for developing an effective program on enhancing dental students’ practice of patient-centered care. The current study supports the idea that education programs should focus on enhancing empathy, emphasizing positive attitudes on learning communication skills, and conducting follow-up educational sessions to prevent students from becoming less patient-centered with an increase in the duration of their academic period.

## Supplementary Information

**Additional file 1.** Questionnaires.

## Data Availability

The datasets used and/or analyzed during the current study are available from the corresponding author on reasonable request.

## Abbreviations

PPOS: Patient–Practitioner Orientation Scale
IRI: Interpersonal Reactivity Index
CSAS: Communication Skills Attitude Scale
PAS: Positive Attitudes Scale
NAS: Negative Attitudes Scale
OECD: Organization for Economic Cooperation and Development
