# Supplementary material for: Empathy and attitude toward communication skill learning as a predictor of patient-centered attitude: a cross-sectional study of dental students in Korea
Source: BMC Med Educ. 2021 Apr 21;21:225. doi: 10.1186/s12909-021-02674-z (PMC8058758; doi:10.1186/s12909-021-02674-z)
Supplement: Supplementary file 1 — Additional file 1. Questionnaires. [file 12909_2021_2674_MOESM1_ESM.docx]

Questionnaires

***Patient–Practitioner Orientation Scale (PPOS)***

Haidet P, Dains JE, Paterniti DA, Hechtel L, Chang T, Tseng E, Rogers JC. Medical student attitudes toward the doctor–patient relationship. Med Edu. 2002;36:568-74.

Krupat E, Yeager CM, Putnam S. Patient role orientations, doctor-patient fit, and visit satisfaction. Psychol Health. 2000;15:707-19.

***Interpersonal Reactivity Index (IRI)***

Kang I, Kee S-W, Kim S-E, Jeong B-S, Hwang J-H, Song J-E, Kim J-W. Reliability and validity of the Korean-version of Interpersonal Reactivity Index. J Korean Neuropsychiatr Assoc. 2009;48:352-8.

***Communication Skills Attitude Scale (CSAS)***

Anvik T, Gude T, Grimstad H, Baerheim A, Fasmer OB, Hjortdahl P, Holen A, Risberg T, Vaglum P. Assessing medical students' attitudes towards learning communication skills–which components of attitudes do we measure? BMC Med Educ. 2007;7:4.
